# Editorial: Exploration of innovative strategies focusing on advanced nanobiomaterials for optimizing oncological treatment and tissue restoration

**DOI:** 10.3389/fbioe.2023.1274758

**Published:** 2023-08-15

**Authors:** Xing Zhang, Johannes Greven, Long Bai, Weifeng Lin

**Affiliations:** ^1^ Department of Orthopedics, Trauma and Reconstructive Surgery, University Hospital RWTH Aachen, Aachen, Germany; ^2^ Institute of Translational Medicine, Shanghai University, Shanghai, China; ^3^ School of Chemistry, Beihang University, Beijing, China

**Keywords:** innovative strategies, versatile nano-/biomaterials, oncological treatment, tissue restoration, biological mechanism, precise nanomedicine, regenerative medicine, osteogenesis

Recent progress in the nanotechnology and biotechnology has sparked a revolution in oncological therapy and tissue restoration, which led to a variety of brand-new nano/biomaterials with distinct structural and functional properties. The emergence of these “smart” nanoplatforms and biomaterials opens up a promising window to address the imperfections of conventional methods in cancer therapy and tissue regeneration, which has greatly improved therapeutic efficacy and accuracy. Exploration of innovative strategies to refine the use of nano/biomaterials is essential to fully exploit their potential.

The Research Topic “*Exploration of innovative strategies focusing on advanced nano-/biomaterials for optimizing oncological treatment and tissue restoration*” was conceived as a Research Topic and reviews spotlighting innovative strategies to potentiate the efficacy of oncological therapy and impaired tissue regeneration with the aid of nanoplatforms and biomaterials. Excitingly, a total of 17 articles have been collected with a well-balanced ratio: 2 comprehensive reviews and 15 original research articles. Herein, we provide a brief synopsis of the articles included in this Research Topic: one review summarizing the recent development of hydrogels for treating bone-related diseases, one review reporting recent advancements in nano-drug delivery for synergistic antitumor immunotherapy, and fifteen original articles presenting the latest advances in the investigation of nano-/biomaterials in the field of cancer treatment and tissue engineering. Our goal is to share innovative solutions to surmount current challenges and hurdles, which shed light on future directions of nano-/biomaterials-based disease interventions and treatment.

In recent years, immunotherapy has emerged as a powerful therapeutic strategy for treating cancer, and its advances have resulted in great improvements in clinical cancer outcomes. In this respect, Zhao et al. systematically review the latest developments in nano-drug delivery systems, classification of cancer immunotherapy, and recent progress in synergistic antitumor immunotherapies by application of nano-drug delivery systems. Nanodrug delivery systems-mediated cancer immunotherapy significantly alleviates the problems associated with immune tolerance and escape, immune side effects and poor tumor targeting, *etc.*, by enhancing their stability and lengthening the circulation time.

To improve the therapeutic efficacy against solid tumors, hydrogel systems integrating various treatment modalities have been developed well. Tang et al. construct CuS nanoparticles and camptothecin (CPT) co-loaded thermosensitive injectable hydrogel (SCH) with self-supplied H_2_O_2_ capacity for enhancing chemodynamic therapy (CDT) in cancer therapy. When injecting SCH hydrogel into tumor sites, it achieves the synergism of photothermal therapy, precise drug release, and self-supply of H_2_O_2_, generating massive hydroxyl radicals and remarkably inducing tumor cell apoptosis. Sulfur dioxide (SO_2_) gas therapy is an emerging therapeutic modality, holding great potential in treating multiple diseases. Huang et al. propose a SO_2_ prodrug doped FeGA nanoparticles for enhanced CDT by photothermally triggered gas therapy (FBH) system, which is synthesized by wrapping a ratio of benzothiazolyl sulfonate (BTS) and FeGA NPs in the heat-sensitive hydrogel. This system can accurately control the release of SO_2_ gas by virtue of the excellent photothermal conversion ability of FeGA NPs. The yielded SO_2_ could induce cancer cell apoptosis but also consume excess GSH and increase the level of reactive oxygen species for improved therapeutic effect of the Fenton reaction. Lung cancer is also tackled in an original research article by Ning et al., developing a CLH hydrogel system that amplifies oxidative stress through cascade catalysis by co-loading CuS nanoparticles and β-lapachone (Lap) into agarose hydrogels. This CLH-mediated reactive oxygen burst strategy is expected to enhance the efficacy of antitumor therapy and reverse tumor chemoradiotherapy resistance in the future. In addition, Wang et al. design a copper-based nanozyme (CuP) and loaded it into agarose hydrogel to form CuP-based hydrogel system (named as CH) for second near-infrared (NIR-II) imaging and breast tumor treatment. This nanosystem could reduce the antioxidant potency of tumors and produce highly toxic OH for destroying the redox steady state, ultimately improving the effect of radiotherapy (RT). Together, these four contribution articles provide an outlook on the translation of “smart” hydrogel system-based strategy into real clinical applications.

Sonodynamic therapy (SDT) is a non-invasive emerging approach based on the interaction of ultrasound and sonoactive substances (sonosensitizers) for oncotherapy. Unfortunately, its clinical outcome is still unsatisfactory owing to the tendency to aggregate and weak targeting ability. In this context, Li et al. present a novel composite ECaC nanosystem by coating porous CaCO_3_ NPs and Cur with the tumor cell-derived exosomes. Tumor cell-derived exosome membranes endow CaCO_3_ with a new immune evasion ability, which enables it to actively evade the clearance of organs such as the liver and kidney, and specifically targets the tumor site to facilitate the release of Cur and Ca^2+^ in response to the degradation of tumor acidic microenvironment. Wang et al. construct a novel nanoplatform by loading Pyrogallol (PG) into mesoporous organosilica nanoparticles for cancer treatment, aiming at boosting intracellular and mitochondrial ROS levels, depleting glutathione (GSH), and downregulating GPX4. Particularly, MON@PG nanoplatforms were proven to significantly inhibit tumor proliferation and growth *in vitro* and *in vivo* by inducing ferroptotic cell death to sensitize radiotherapy. Interestingly, this work offers a promising chemoradiotherapy regimen for gastric cancer treatment by disrupting redox balance and augmenting ferroptosis. Bao et al. design the ferrimagnetic vortex nanoring Fe_3_O_4_@HA (FVNH) nanoparticles enclosing Doxorubicin for T2 MR imaging-guided hyperthermia therapy and chemotherapy. Notably, FVNH NPs show significantly extremally high magnetothermal conversion efficiency and shoring the transverse relaxation time for enhanced MRI imaging. Overall, FVNH loading with DOX nano-construct might hold great potential for combined magnetothermal-chemotherapy and targeted cancer imaging in future clinical trials.

Deep vein thrombosis (DVT) is a common cause of disability and cardiovascular death worldwide. Chen et al. realize the diagnosis and treatment of thrombus by applying low-power ultrasound combined with targeted microbubbles (MBt) bearing TNF-α antibody and urokinase (United Kingdom) in a DVT animal model. TNF-α is used as the target of thrombus to attract numerous targeted microbubbles to enrich locally in the thrombus and neutralize the inflammatory response, presenting an innovative strategy to treat thrombus, which is in line with the theme of our Research Topic. Lymphedema is a progressive disease caused by lymphatic transport dysfunction, imposing a substantial biomedical burden. Liu et al. reveal the relieving effect of low-intensity pulsed ultrasound (LIPUS) on secondary lymphedema, but also verify that LIPUS reduces lymphedema by regulating macrophage polarization and enhancing microcirculation. Accordingly, this innovative work offers a promising way for lymphedema treatment. Research conducted by Wang et al. delineates three primary aspects of nanoparticle-leukemia-related studies, including nanoparticles for diagnosis and treatment of leukemia, the specific molecular mechanism, and existing problems of the application of nanoparticles in leukemia. This offers preliminary and objective insights into nanoparticle-mediated leukemia studies.

Liu et al. contribute to this Research Topic with a review summarizing the evolution, properties, and preparation methods of hydrogels, and their latest application in bone-related diseases involving bone defects, fractures, cartilage damage, and osteosarcoma. The authors point out that hydrogel has great application potential as a functional polymer material in bone tissue engineering due to its unique advantages, such as porous structures similar to the extracellular matrix (ECM) and the soft texture reducing surrounding inflammatory responses. This review proposes customized suggestions for developing high-performance hydrogels suitable for bone-related diseases.

An excellent example of extracellular vesicles (EVs) for bone defect treatment is presented by Zhang et al., who develop dexamethasone-stimulated osteoblast-derived EVs (OB-EV_Dex_) and investigate their osteogenic potential as a novel biomimetic tool to accelerate bone augmentation. The authors demonstrated that OB-EV_Dex_ could markedly promote osteoblastic differentiation by positively upregulating crucial osteogenic genes, but also significantly augment capacities for *in vitro* proliferation, attachment, and viability of osteoblasts. Of note, the pro-osteogenic effect mediated by OB-EV_Dex_ was even comparable to that of individual BMP-2 treatment. It is reasoned that irrespective of any clinical challenges, the prospective use of OB-EV_Dex_ could function as a novel osteogenic accelerator for treating, or at least alleviating the symptoms of bone defects in future clinical practice.

Du et al. report a bifunctional Bi-BG composite scaffold with excellent photothermal effect and osteogenic activity, making it possible to treat large bone defects caused by surgical resection of bone tumors. This innovative strategy based on the Bi-BG scaffold allows the photothermal ablation of bone tumors and the augmentation of osteogenic capacity, which realizes the aim of “one stone, two birds”. This work may provide new ideas for treating and repairing tumor-associated bone tissue defects by developing novel bifunctional scaffolds. Feng et al. propose a NIR light-assisted strategy to treat infected wounds based on the newly synthesized photothermal agent of CuSi nanowires, which could not only effectively combat bacteria, but also promote angiogenesis and facilitate wound healing due to the sustained release of bioactive Cu and Si ions. Qiang et al. contribute to this Research Topic with an original article reporting a high-strength Zn0.8Mn0.1Li alloy with calcium-phosphorus coatings for orthopedic implants. Such a self-designed implant significantly satisfies the clinical demands associated with improved osteogenic ability, biocompatibility, and biodegradability of medically degradable metal materials. The proper microenvironment is critical for the storage and transportation of embryonic stem cells (ESCs). Yang et al. propose an alternative approach that allows for facile storage and transportation of stem cells in ESCs-dynamic hydrogel construct (CDHC) under ambient conditions. This dynamic and self-biodegradable hydrogel provides a simple, cost-effective, and valuable tool for storing and transporting “ready-to-use” stem cells, facilitating “off-the-shelf” availability and widespread applications in biomedical fields and regenerative research.

In conclusion, this Research Topic presents state-of-the-art research work centering around the topic of nano-/biomaterials-based innovative strategies for oncological therapy and tissue restoration. There is a consensus among the authors that attention should be dedicated to optimizing the structure and properties of nano-/biomaterials for maximizing therapeutic efficacy. This Research Topic also provides distinct insight into overcoming the existing hurdles and challenges, and future directions associated with the design and development of these nano-/biomaterials. We hope this Research Topic could be of great interest to researchers and professionals, ultimately advancing advanced nano-/biomaterials towards clinical translation and improving clinical therapeutic outcomes.

